# How older adults recovering from substance use problems experience mattering

**DOI:** 10.1186/s12913-023-10413-x

**Published:** 2023-12-21

**Authors:** Nina Kavita Heggen Bahl, Hilde Eileen Nafstad, Rolv Mikkel Blakar, Emil Øversveen, Morten Brodahl, Ottar Ness, Isaac Prilleltensky

**Affiliations:** 1 Department of Research and Development, Clinic of Substance Use and Addiction Medicine, St. Olavs hospital, Trondheim University Hospital, Klostergata 48, 7030 Trondheim, Norway; 2 Department of Psychology, University of Oslo, Forskningsveien 3A, 0373 Oslo, Norway; 3 Department of Sociology and Political Science, Norwegian University of Science and Technology, 7491 Trondheim, Norway; 4 Norwegian National Advisory Unit On Concurrent Substance Abuse and Mental Health Disorders, Mental Health Division, Innlandet Hospital Trust, Divisjon Psykisk Helsevern, 2381 Brumunddal, Norway; 5 Department of Education and Lifelong Learning, Norwegian University of Science and Technology, 7491 Trondheim, Norway; 6 School of Education and Human Development, University of Miami, Coral Gables, Florida, USA

**Keywords:** Later life, Mattering, Recovery, Substance use problems, Qualitative methodology

## Abstract

**Aim:**

Mattering (to feel valued and add value to self and others) is a fundamental human experience and mechanism in recovery. In this paper, we concern ourselves with the recovery of older adults with substance problems. This population is on the rise in many Western countries. To offer mattering enhancing programs for this group, more knowledge about later life mattering in service-assisted recovery processes is needed. This study aims to explore experiences of mattering in older adults receiving services to recover from substance use problems.

**Methods:**

A collaborative and deductive reflexive thematic approach was applied in analysing 23 interviews with participants using substance use services. Participants were recovering from different substance use problems: alcohol, medication and illegal substances. The participants were recruited from three different Norwegian social contexts: two urban and one medium size municipality. The age of the sample ranged from 65–80 years, with approximately equal numbers for those aged 60–69 (12 participants) and 70–80 (11 participants). Seven participants were women and 16 men.

**Results:**

Three main themes were identified in the analysis: “relational experiences of mattering and not mattering”, “service-related experiences of mattering and not mattering” and “recovery and psychological sense of community as interrelated phenomena to experiences of mattering”. The findings illustrate various nuanced experiences of mattering and not mattering in later life recovery processes.

**Conclusions:**

Overall, the participants’ mattering experiences rested on fair, healthy and positive community relationships and fair and attentive services, where participants could feel valued and also have a chance to add value to others. Experiences of *not mattering* were precipitated by lack of support, disrespect, devaluation and loss of relationships, and also by being ignored and not receiving fair treatment and help by professionals. Importantly, reciprocal and enhancing relations between mattering, recovery and relational PSOC seem to exist and to be significant for the older adults’ access to substance use services. Several practical implications are suggested to promote the therapeutic and preventive potentials of later life mattering in recovery.

## Introduction

To thrive and have a good life, we all need to experience social worth, dignity, and respect, regardless of age, gender, race, ethnic identity, geographical location, socioeconomic or occupational status [1–3]. Mattering is a fundamental human experience across the life span, often experienced in communities. Conceptually, mattering can be defined as a state in which people “feel valued by, and add value to, self and others”. The experience of mattering is also highly correlated to positive psychological outcomes, including happiness [4], meaning [5], overall well-being [2, 6], workplace well-being [7], and life satisfaction [8, 9]. A life lacking in mattering is associated with suicidal ideation and attempts [10–13], depression [14, 15], aggression [3, 16], loneliness [17] and overall stress [18], and usually takes place when we feel marginalized, excluded, devalued, and disrespected [2].

Despite increasing investigations of mattering and its relevance in promoting health in later life, older people have, until recently, been largely ignored in studies of mattering. In a recent review of mattering in old age, however, Flett and Heisel [19] argue that mattering is a unique protective factor in the prevention of mental and physical problems among older adults. Their review also suggests that later life mattering can promote crucial components in recovery processes, such as the experience of belongingness and a meaningful life [19, 20]. Older adults with substance use problems (defined as 65 year <) is a group on a steep rise in several Western societies [21–23]. Consequently, there is a pressing need today for substance use services tailored for this group [23–25]. More knowledge about later-life mattering from older adults in recovery processes assisted by substance use services is needed to tailor and offer services promoting mattering for this population. Focusing on first-person accounts from older people using different substance use services to assist their recovery processes from substance use problems, we explore experiences of mattering and propose some implications for action for substance use services.

### Theoretical Framework: The Role of Mattering in Recovering from Substance Use Problems in Later Life.

Mattering is about a balance between feeling valued and adding value, to self and others, throughout one’s life [19, 26]. The fulfilment of this balance depends on the particular social context of community members [27]. Moreover, *the domains* where people experience feeling valued and/or adding value may change across the lifespan. This seem to be the case also for older adults with substance use problems, although we know little about this group.

Relevant studies suggest that individual resources (e.g., health), social resources, communities, health care services, and meaningful activities are central ingredients to mattering for people with substance use problems [25, 28–31]. A very important factor in mattering is psychological sense of community (PSOC), which has been defined as ‘the perception of similarity to others, and acknowledged interdependence by giving to or doing for others what one expects from them, the feeling that one is part of a larger dependable and stable structure’ [32]. This factor has been identified as a central dimension in later life recovery [33, 34]. These findings are in line with other studies on relational recovery [31, 35]. Relational recovery focuses on recovery as a social process [36, 37] that addresses structural factors (e.g. poverty and housing) [38], as well as individual aspects of personal recovery [31]. Furthermore, relational recovery emphasises that personal experiences of recovery depend on the relationships and social contexts of the person [31], rather than something that happens to and within an individual [37]. This understanding of recovery as a relational process builds on a view of persons as fundamentally relational beings [39]. According to this literature, the experience of mattering in recovery processes, most likely, depends on relationships and the social context, also for older adults recovering from substance use problems.

Substance use services are a central part of the context for older adults in service-assisted recovery processes. In these services, user involvement and shared decision making can enhance the sense of mattering. User involvement and shared decision-making processes are well-established in the consumer/survivor movement. This is a movement created by patients who were part of the psychiatric system and that took control of their fate. Instead of relinquishing control of their illness primarily to the medical establishment, consumer/survivors became empowered and insisted on leading the treatment plan, as opposed to passively acquiescing to orders from psychiatrists, psychologists, nurses, and social workers. Some of the principles advanced by consumer/survivors include independence, empowerment, a member-driven approach, and more egalitarian relationships among caregivers and recipients [40]. This approach has been widely disseminated and is now used and recommended in the treatment of vulnerable populations afflicted by homelessness, substance abuse, psychiatric conditions, health challenges and oppressive conditions [41]. Mattering, empowerment, and shared decision-making fall under the umbrella of the SPEC approach; according to which service seekers and recipients expect treatment plans to be guided by the principles of Strengths, Prevention, Empowerment, and Community change [42, 43].

However, the existing evidence of mattering in later life, mattering in recovery from mental health issues and mattering for service providers provides little theoretical insight to the subject matter. There is a need to understand better the experiences of mattering for older adults recovering from substance use problems. Findings suggest that when psychosocial transitions in later life are complicated by substance use problems, the struggle to achieve mattering may become even more difficult and complex due to the increased likelihood of factors such as deteriorating health, comorbidity, depression, shame, loneliness and social isolation [25, 44–47]. Methodologically, the most relevant research on the subject has either used quantitative approaches or qualitative research leaving out the “expertise by experience” in the analysis of data. Thus, although there is some evidence suggesting potential resources and obstacles for mattering in later-life recovery processes and the importance of mattering in service-assisted recovery, there is, to our knowledge, no existing empirical research on experiences of mattering or not mattering in later-life recovery from substance use problems. Furthermore, there is no existing research on the subject matter including a peer perspective to understand these experiences from a service user perspective.

Considering the unique protective and promoting psychological effects of later life mattering (e.g., self-esteem, social support, lower depression and greater psychosocial well-being and wellness) [19, 48], more knowledge about mattering among older adults with substance use problems is essential for substance use services. Specifically, there is need for knowledge about mattering from older adults in service-assisted recovery processes to enhance and offer the therapeutic and preventive implications of mattering for this particular and growing population. Older adults recovering from substance use problems have lived experiences of mattering and not mattering which provide crucial information to understand the complex and under-studied topic of mattering in later life recovery. Thus, there are currently theoretical, empirical, methodological and service-related arguments for an investigation of the mattering experiences of older adults in recovery from substance use.

### Current study

Qualitative research provides the opportunity to explore under-studied topics in-depth from the perspective of the population of interest. This qualitative and exploratory study aims to explore and understand experiences of mattering for older adults in service-assisted recovery processes from substance use problems. By a collaborative research approach including a community psychological, sociological and peer point of reference [49–51], we assume that older adults recovering from substance use problems are social agents and experts on their own lived experiences. They understand best their conditions promoting and challenging experiences in their personal recovery processes. Furthermore, we have taken a reflexive, deductive thematic orientation [52–54], assuming that theoretical frameworks, also the framework of mattering [19, 26], can be used as a relevant theoretical lens to move beyond obvious meaning in the data, thereby guiding, informing and deepening our collaborative analysis and interpretations of the participants’ experiences.

To elaborate, in line with a *reflexive* thematic analysis (see [55]), the researchers’ perspectives were integrated as resources in the deductive analysis of older adults’ experiences of mattering. Quality in reflexive thematic analysis is about the researcher’s reflexive and thoughtful engagement with the analytic process of constructing themes from data. If multiple researchers are involved in the analytic process, the approach is collaborative and reflexive, aiming to develop a rich and nuanced construction of themes, rather than seeking consensus on meaning [55]. Thus, with a shared theoretical framework as reference, this study used, integrated and merged three different points of views from different researchers in the co-construction of themes for a nuanced and in-depth understanding of older adults’ experiences of mattering. To provide transparency of the researchers’ perspectives, we have included a note on “Researchers’ perspectives” for the researchers involved in the initial stages of the analysis.^1^

We have analysed data from a national Norwegian qualitative study on service user experiences of older adults struggling with substance use problems and receiving municipal services for persons with substance use problems (see [56]). The data included 23 interviews with participants (7 women, 16 men) from different Norwegian municipalities having different substance use problems: alcohol, medication and illegal substance use. 21 of 23 interviews included descriptions of feeling valued by or adding values to self and others. Guided by the participants’ descriptions of mattering, we have explored older adults’ experiences of feeling valued by, and adding value to, self and others during their service-assisted recovery processes.

## Methods

### Sample and recruitment

As presented, this study utilized data from a national project evaluating service users’ experiences with substance use treatment services from Norwegian municipalities (see [56] for further details). The Drug and Alcohol Competence Centre in Central Norway conducted this study on assignment by the Norwegian Directorate of Health. The national project’s second wave aimed to generate qualitative knowledge about how older adults with substance use problems experience the different substance use services offered from the Norwegian municipalities.

A purposeful sampling strategy was used in the study to recruit 23 older adult participants with substance use problems from three different contexts: two urban and one medium size municipality. The age of the sample ranged from 65-80 years, with approximately equal numbers for those aged 60-69 (12 participants) and 70-80 (11 participants). Seven participants were women and 16 men.

Different groups of service staff (e.g., geriatric psychologists, staff at user organizations, and substance use treatment clinics) working with older adults with substance use problems collaborated and assisted in the planning of participant recruitment in all three contexts in 2019 (prior to the Covid-19 pandemic). Staff contacted potential participants directly by phone and in physical meetings, as well as contacting services relevant for recruitment of additional participants (e.g., general practitioners, home nursing, low threshold services where no formal referral is required (e.g., the Salvation Army’s social security and welfare services and contact centres helping with basic needs such as nutrition, clothing, and hygiene) and geriatric clinics in specialized health care). The services were contacted in physical meetings, and by email, phone, and newsletters inviting them to recruit participants.

All participants recruited had to meet the following criteria: age 65 years or older (which is consistent with definitions of old age in populations with substance use problems [45, 57]; as well as having a substance use problem with alcohol, medicine or illegal drugs and thereby receiving assistance from one or several substance use services from the municipality in which they resided. Overall, we had a heterogeneous sample of older adults recovering from substance use problems (see Table 1).

**Table 1:** Participants’ background

**Participant**^a^	**Interviewer**^b^	**Region**	**Services received**	**Current use of substances?**	**Onset of substance use problems**	**Way of contact with services**	**Problematic substances**	**Available relational communities**
M66	5	East	Housing, housing allowance, NAV, organized physical exercise, general practitioner, low threshold offer (meals)	No	Early	Employer made contact	Amphetamine/Alcohol (Polysubstance use)	Family Friends
M67	4	East	Housing, contractual early retirement pension scheme (AFP) in the public sector, earlier: three different institutions, home nursing,	No	Early	Injury/hospitalization	Opioids/Alcohol (Polysubstance use)	Family Friends
M77	5	East	NAV, disability pension,	Yes	Early	Lack of income	Alcohol	Family Friends
M71	2	West	Contractual early retirement pension scheme (AFP) in the public sector, general practitioner, earlier: institution, NAV Assistive Technology Centre, institution	No	Early	Self-initiated contact	Alcohol/sleeping pills (Polysubstance use)	Family Friends
F68	5	East	Housing, Specialized health care (somatic) after injury, AV, Work assistance allowance (AAP), transport service card, general practitioner, physiotherapy, Earlier: six institutions	No	Early	Suicide attempt/hospitalization	Alcohol/Morphine (Polysubstance use)	Family Friends
M68	2	West	NAV, Crisis centre for victims of violence and abuse from partner or family, contractual early retirement pension scheme (AFP) in the public sector, organized physical exercise, earlier: institution	No	Early	Violence in the home (partner)	Alcohol	Family Partner Friend
M80	4	East	Housing, pension (not specified), outreach service, home nursing, earlier: institution	No	Early	Recommended by family to make contact	Alcohol	Family
M77	1	Central	Pension (not specified), home nursing, housing, NAV Assistive Technology Centre, general practitioner, geriatric psychologist, Earlier: recovery centre, physiotherapy,	No	Late	Recommended by home nurses to make contact	Alcohol	Family Friends
M76	1	Central	Pension (not specified), home nursing, NAV Assistive Technology Centre, earlier: institution, general practitioner	Yes	Very late (after 60 years)	Recommended by home nurses to make contact	Alcohol	Family
F65	1	Central	NAV, contractual early retirement pension scheme (AFP) in the public sector, earlier: organized physical exercise for chronical illness, follow-up service, centre for mapping and follow‐up	No	Late	Chronic muscular pain lead to hospitalization	Alcohol	Family Friends
M68	1	Central	Pension (not specified), physiotherapy, home nursing, earlier: institution (specialized health care),	No	Late	Friend assisted in making contact	Alcohol	Friends
F68	1	Central	Housing, contractual early retirement pension scheme (AFP) in the public sector, earlier: institution,	No	Early	General practitioner made contact	Alcohol	Family Friends
F73	4	East	Pension (not specified), organized physical exercise, general practitioner, short-term specialized treatment of alcohol addiction, earlier: institution (twice), home nursing	Yes	Late (20 years)	Injury (fall)/hospitalization	Alcohol	Family Friends
M76	4	East	Pension (not specified), non-governmental organization for persons with alcohol dependence (Norske lenker), nursing home, physiotherapy, social worker, earlier: institution	No	Early	Injury (fall)/hospitalization	Alcohol	Family Friends
F66	4	East	Housing (municipality), psychologist, home nursing, disability benefits, drug-assisted treatment, interdisciplinary team meetings Flexible Assertive Community Treatment, NAV (economic manager), general practitioner, transport service card, earlier: institution psychiatric/substance use	No	Early: Medicine, Late: Heroin (40 years old)	Death of husband who was co-addict	Medicine, heroin, amphetamine (Polysubstance use)	Family Friends
M66	5	East	Housing, disability benefits, drug-assisted treatment, NAV, general practitioner, earlier: institutions	No (methadone)	Early	Personal contact at NAV made contact	Heroin	
M69 (E17)	5	East	Pension (not specified), NAV (management of economy), department of mental health, general practitioner, interdisciplinary team meetings home nursing, earlier: institution, cancer nurse,	No	Early (not specified, but had problems in working age)	Self-initiated contact with hospital due to suicide ideation	Alcohol	
M69 (E18)	5	East	Housing, drug-assisted treatment, general practitioner, old-age pension, polyclinic treatment for persons with substance use problems where alcohol is the only or dominant problematic substance, low-threshold health and care offer for those with substance use problems, social worker, interdisciplinary team, earlier: psychologist, institution	No	Early	Quit work to become clean (self-sufficient), made contact due to starvation	Heroin, alcohol, amphetamine (Polysubstance use)	Family
M69 (C19)	1	Central	Housing, home nursing, social worker, centre for mapping and follow‐up,, district psychiatric centre, earlier: institutions	No	Early	Made contact due to suicide ideation	Alcohol, medicine (Polysubstance use)	Family
M70 (C20)	1	Central	Nursing home, pension (not specified), NAV (economic management), general practitioner, social worker, earlier: institutions (detoxification/substance use treatment)	No	Early	General practitioner referred to hospital (acute)	Alcohol	Family Friends
F70 (C21)	1	Central	Home nursing, pension (not specified), psychiatric nurse, general practitioner, physiotherapy, occupational therapy, primary contact (at department of health and welfare), earlier: follow-up service	No	Late (40 years old)	Hospital made contact after operation	Medicine, alcohol (Polysubstance use)	Family Friends
F73 (C22)	3	Central	Old age pension, organized physical exercise, physiotherapist, psychologist, psychiatrist, general practitioner, home nursing, earlier: institution	No	Very late	After hospitalization (acute)	Alcohol	Family
M72 (C23)	1	Central	Old age pension, daily social offer including physical exercise (9–14), geriatric care, general practitioner, home nursing, earlier: institution (detox)	No	Early		Alcohol	Family Friends

^a^Participants are represented with codes indicating their gender (F for female, M for male) and age.

^b^Interviewer 1 is the fourth author and an academic researcher; Interviewers 2 and 3 worked at The Drug and Alcohol Competence Centre in Central Norway, interviewers 4 and 5 worked at The Drug and Alcohol Competence Centre in Oslo. All interviewers had academic training in conducting interviews (5 of 5) and 3 of 5 had clinical competence in communication with individuals with substance use problems.

### Data material

The data for the present study was a verbatim transcribed interview material. By a piloted interview guide (see [56] for further details), participants were asked to describe their background (e.g. age, years of residency in the municipality, living situation, financial situation, health and use of substances), experiences with their current life situation (e.g. social life, health, substance use), their community relationships (e.g., family, friends, and neighbours), and experiences with the municipal services they received. Examples of questions were: “How would you describe your current health?”, “How would you describe your use of alcohol, medication or illegal substances?” “Do you experience that you get the help you need from your services?”, “If you have any next of kin, friends or other people involved in the assistance from your services, what meaning has this had for you?”, “Do you experience having an co-determination in your services?” and “If you could get just the assistance that you need, how would that service offer look like?”.

### Substance use services and sociocultural context

The social and cultural context of this study was Norway, a Scandinavian welfare state. In the Norwegian public health care system, specialist health care services are offered at the regional level and primary health care services are organized and delivered by the municipalities. As part of Norwegian clinical substance use treatment, individuals are first offered primary services by the municipality prior to and after specialized treatment. Aside from an initial excess charge of NOK3040 per year (approximately USD275), all services are offered free of charge.

Generally, individualistic Northern counties have had less of a family orientation compared to Southern and Eastern European countries [58]. There are studies indicating that family and friends are important social resources in later life recovery processes from substance use [59]. However, given the welfare system and increasing individualistic orientation and meaning systems in the Norwegian context [60, 61], there can be lower expectations of family support and higher expectations of support from services in the recovery processes than in more market-driven and familial and collectivistic contexts.

### Approach to enquiry

This study applied an explorative, collaborative deductive and reflexive thematic approach. The collaborative approach defined the overall study and collaboration with persons who were experiencing, or had experienced, service-assisted recovery took place in all phases of the research project. First, a peer support worker from the Drug and Alcohol Competence Centre in Central Norway participated in the planning of the data collection, piloting and development of the semi-structured interview guide. Second, the data included the perspective of 23 older adult participants holding different experiences of service-assisted recovery processes. Third, in the data analysis, a peer researcher (fifth author) collaborated with the first and fourth author in the analysis to enhance reflexivity and interpretive depth. This peer researcher also had personal experience with recovery assisted by services, work experience with people in recovery from substance use, as well as research experience with qualitative collaborative research within the field of substance use and addiction.

With respect to dimensions for thematic analysis, the analysis was as presented deductive in its theoretical approach, epistemologically experiential, as well as constructivist in its perspective [52–54]. To elaborate, the theoretical framework of mattering was used as a conceptual framework in coding and producing themes. A central experiential assumption, furthermore, was that that the participants’ descriptions mirrored their spoken experiences. Finally, the study is social constructivist as the multi-perspective analyses moves beyond the experienced phenomena of mattering to understand how mattering is socially constructed by community, relationships and services for persons with substance use problems.

### Data analysis

As introduced, there are currently theoretical, empirical, methodological and service-related arguments for conducting a qualitative and explorative study on mattering experiences in later-life recovery from substance use problems. Having data from 23 older adult participants describing their experiences of service-assisted recovery, the first author had the idea of exploring the material with respect to the mattering concept. The idea came initially from a previous analysis of the material (see [56]), which gave the first author the impression that the concept could be central in capturing some of the older adults’ experiences. The first approach was to invite the fourth and fifth authors to undertake a collaborative and deductive thematic analysis of mattering. The first author made clear her observation and interest in conducting an explorative and collaborative study of the subject matter. The invited authors agreed to undertake the proposed analysis with mattering as the guiding theoretical framework, searching for: “experiences of feeling valued by, and adding value to, self and others” [62]. Then, the first author undertook a coding of the material according to her community psychological perspective. The coding showed that 21 of 23 interviews included descriptions of mattering and with a total of 216 coding references to mattering. These coding references were used in the first authors search for themes. Parallel, the fourth author conducted his individual coding and search of themes according to his sociological perspective, while a peer researcher (fifth author) systematized potential patterns and insights about mattering from a peer perspective, from the overall material – also guided by the shared theoretical framework. Thus, the coding and search for themes was conducted in a wide manner, including and analysing all utterances, which to each of the researchers’ perspectives - community psychological, a sociological and a peer perspective- could be understood as reflecting a mattering experience.

**Fig. 1:**
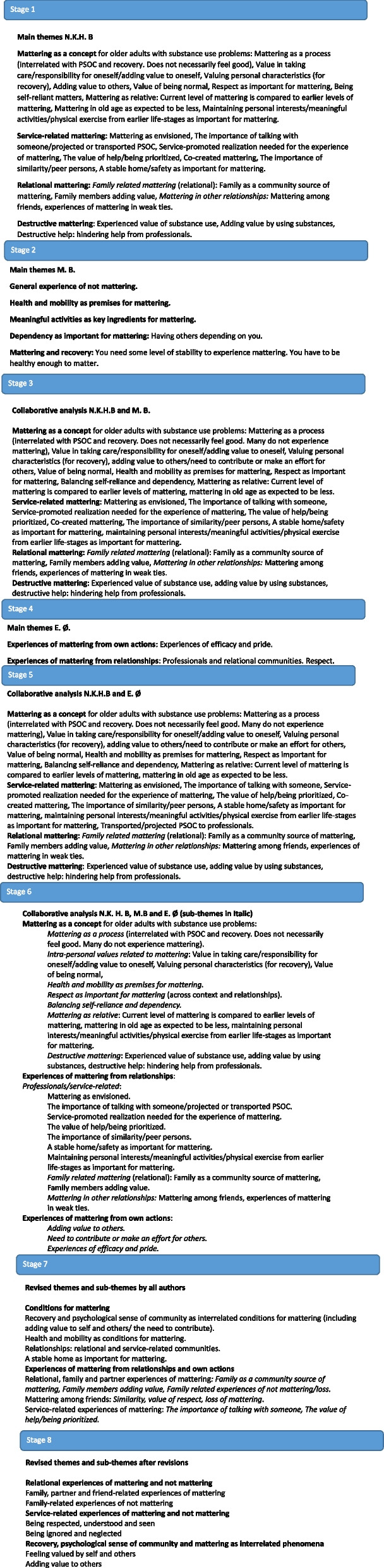
Development of themes

Overall, the analysis was conducted through eight stages (see Fig. 1). The reviewing, defining, and naming of themes were carried out collaboratively in stage three to seven. Furthermore, the development of themes involved three collaborative analyses, conducted by dialogue, reflection and discussions about the theme introduced by each of the researcher’s perspectives. The aim of these meetings were not to reach a consensus, but rather to voice, integrate and merge all perspectives in the co-creation of themes. The two first collaborative analyses were undertaken by the first author and each of the other two researchers, while the third analysis was done by all three researchers. The first author made sure to systematize and merge all of the researchers’ themes in stage three to six. In the seventh stage of the analysis, all authors agreed on the final themes for the article. Finally, an eighth and final alteration of themes was done as part of the revisions of the article. Consistent with the revisions, themes were narrowed down to and systematized with respect to *experiences* of mattering in service-assisted recovery processes.

## Results

Three main themes were generated from the analysis: “relational experiences of mattering and not mattering”, “service-related experiences of mattering and not mattering” and “recovery, psychological sense of community and mattering as interrelated phenomena.” As can be seen in Fig. 1 (stage 2), there was a common experience for several participants of *not mattering*. These participants described experiences of not being worthy of services or help, feeling strong guilt related to their life with substance use problems. To gain a broad and needed understanding to enhance the therapeutic and preventive implications of mattering, we will focus on experiences of both mattering and not mattering.

### Relational experiences of mattering and not mattering

Relational communities such as family, partner and friends are central sources for mattering experiences [62]. When it comes to older adults, there are findings suggesting that older adults experience mattering primarily to their children [48]. This was reflected in seven of the participant descriptions of family- related mattering as important in initiating a service-assisted recovery process. Experiences of being valued by one’s grown up children and partner were often connected to receiving help and support from services in their process of recovery:

P^2^: And, then the fight (for help) started. Because there wasn’t any offer that wanted to take me in. So, then I said that “I have to get an offer now”. And my son said the same. “If not, she is going to die”.

I: So, your son came to the rescue?

P: Yes, he supported me.

I: What meaning has it had for you that the family and your children have been involved in the recovery process?

P: That has meant everything to me. If I had been alone in that process, then….I’m not so sure I would be here today. Then I don’t think I would have felt that I mattered or had anything to live for, frankly.

I: Can you elaborate on that?

P: Just that you gain your courage to live. Because that was completely gone (in the beginning of the process).

Common for the examples in the material was that they illustrated that the experience of mattering to one’s family promoting the service assisted recovery process; and as a consequence, outcomes such as illness management and courage to live seemed to strengthen the experience of mattering between the older adults and their family.

Family can promote both protective (e.g., support and positive PSOC) and inhibiting factors (e.g. trauma and exposure to substance use) through recovery processes [33, 34, 59]. In the current analyses also, families seemed to have a mixed role, both as a source of mattering and not mattering for the recovering older adults. Experiences of disrespect, devaluation as well as loss of family or partner-related mattering trough death were central nuances to the theme. The following is an example of how families could promote central elements of not mattering, such as disrespect and devaluation:

…Once I celebrated Christmas with a close relative and her children when they were still little children. I had brought gifts for them, and their mother (his relative) had opened their gifts and decided that they should not have those gifts and thrown them in the bin. So, when I asked the children if they liked the gifts, they just looked at me as question marks. I have no words. It was so rude! After that, I didn’t want to celebrate Christmas with my family.

Persons with substance use problems often have strained relationships with their family. Such strains may turn sources of mattering, such as the family, into sources of not mattering. The participants’ descriptions suggested that, just as there can be a fine line between love and hate in families, there can also be a fine line between mattering and not mattering.

Loss of relational mattering through death was another factor that could promote the experience of not mattering in old age; for some resulting in suicide ideation:

P: You know. Sometimes I wonder, what is the point of living? You know (cries)?

I: Can you talk to someone about that?

P: No, I haven’t done that. My partner died many years ago…And I miss him so much….two or three months ago I thought about ending my life. In the mornings I wake up and put my hand over at his side of the bed…then I feel that he is gone…everything has been heavy and empty after he died…

Like for family and partner, loss of friend-related mattering through death was an experience promoting lack of mattering in later life: “We knew each other about 20 years. When he died, I didn’t feel that I mattered anymore”.

Friends were described an important source for mattering in later life recovery and this was evident in the description of three of the participants. Respect and similarity were described as important ingredients of these participants’ friend-related experiences of mattering.^3^ In addition to sharing similar interests and recovery experiences, sharing and confirming similar experiences with services were described as important for claiming experiences of mattering:

…She (his friend) has been there for me (in the recovery process). We both think that one of the staff in the home service is terrible. So, we support each other. Make sure we get what we should have. That makes it easier for me to make claims. That my needs matter.

Once again, the participants’ descriptions suggested the importance of *relational* community relationships for experiences of later life mattering in recovery.

### Service-related experiences of mattering and not mattering

To experience mattering, we must feel that we are respected and seen by others [62]. The older adult participants were all in recovery assisted by services offered to those having substance use problems (see Table 1). Several participants described how important it was to talk *with* service providers to experience mattering, in a way that made them feel that their needs were respected and their problems were understood:

P: I talked with my doctor, and he was very nice. Very welcoming. I got a lot out of taking to him.

I: Can you say a little more about what you mean?

P: Yes, he was very good. I have to say.

I: But if you were to describe what you valued? What was it that he did?

P: I don’t know.

I: Was it that he talked with you?

P: He talked *with* me and not *to* me.

I: And what would you say is the main difference? Of talking *with* and *to*?

P: To talk *to*, it to kind of say «now you will do that and that»…

I: Like a command?

P: Yes. But he didn’t do that. He was welcoming and understood.

I: And, talking *with*. What is that?

P: That he could see my problems. That they mattered and that he would try to fix those problems.

Relatedly, we identified some examples suggesting that in addition to feeling respected and understood, receiving support from service professionals having the skills to identify ones’ values in life, was important to promote the experience of service-related mattering:

P: X (geriatric psychologist) knew that I had tried to end my life, so she had something concrete to deal with. It was evident that I needed to get back on my feet. For a long time, everything was so insecure. I just felt that I and my general practitioner couldn’t make my life matter to me. I ran. Exercised every day and did all that I could (to manage health), but things were just not right. But with X, she made me see the value in things.

I: What significance has that had for you?

P: Very much. Like I matter.

Another central part of the participants’ descriptions of service-related mattering was the value for them of continuing experiencing being seen and prioritized, also in follow-up:

I was admitted to hospital for alcoholism…and I was there for a while, and then I felt that I got a really good follow-up in the way that I was prioritized, or given attention, right? That “you have a need and we will help you”…that mattered a lot to me.

On the contrary, experiences of being ignored by service professionals (e.g. by not having the time to talk, often mentioned with respect to home nurses), promoted service-related experiences of *not* mattering:

I think they could add a little to the conversation. “How are you?” “Are you doing good?” Ask a little bit about those things. I feel that is lacking in these home services. They don’t have the time to talk to people…That gives me a negative experience.

Finally, there were nuances to this sub-theme suggesting that service-related experiences of not mattering were promoted by experiences of service professionals neglecting the needs of those having substance use issues:

I: If you could get any help that you desired (for your recovery)? How would that service look like?

P: Well…I have to base my answer on my own experience. And…that is that you have to be so sick that you are dying (before you get help). It shouldn’t be necessary to fight to get help. In that case the offer should be there. I am aunt of two persons who have taken their life. Both being drug addicts. And they were calling for help but weren’t prioritized. So, I am not impressed (by the services offered).

In this case, support from family members (adult children) came to the rescue for the participant, securing her recovery and mattering. This example also illustrated another pattern—the importance of having supportive relational community relationships when services promote the experience of not being valued.

### Recovery, psychological sense of community and mattering as interrelated phenomena

An overarching theme in the material, permeating the other themes, suggested that recovery and psychological sense of community (PSOC) were interrelated with the participants’ experience of mattering. This interrelation was identified with respect to feeling valued by self and others, and adding value to others. With respect to the former (feeling valued by self and others), recovery seemed to make relational sources of mattering available:

When we (her friends and acquaintances from that place) get together. And people are taking a pint or a glass of wine. At that moment I miss it (drinking alcohol). Luckily, when that thought strikes me, I think that “No. It’s not going to be that way. You shouldn’t have that (alcohol)”. And I appreciate that. That awareness. So that I can be with the people who make me feel that I belong.

As this example suggests, not only did the participant feel valued by friends, she also added value to self by appreciating her new strength in managing the substance use problem. This element of mattering empowered her recovery process, fuelling a reciprocal interaction between the three phenomena. There were similar examples in the material with respect to family:

…Contrary to many others I have maintained a close relationship with my family…And that of course matters a lot to me in my recovery. Because I hear…I speak to others with substance use problems, and they don’t have any contact with their family. They (the family) have been there for me. Not pointing any finger…respecting me.

**Fig. 2:**
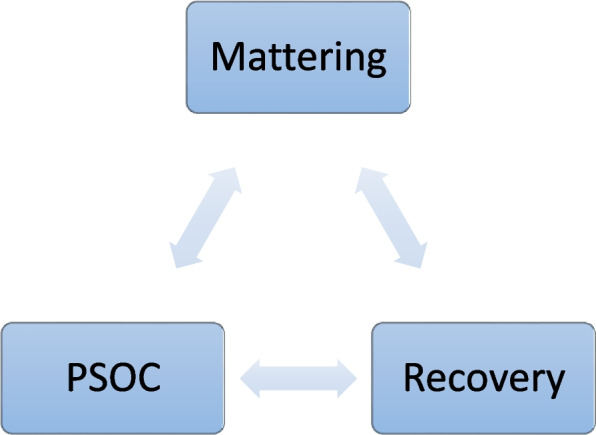
Recovery, psychological sense of community and mattering as interrelated phenomena

From this quote, we see that the participant maintained a family connection and thereby a source of mattering through his process of recovery, assisting his recovery process. As such, PSOC and mattering facilitated the recovery process. However, the examples of interrelations also seemed to suggest that recovery facilitated a stronger PSOC and mattering within the family: when the families as a community had a shared experience of going through a demanding process of recovery together, this seemed to promote the older adults experience of mattering to the family. Again, we see the reciprocal relations between recovery, PSOC and mattering, and their way of enhancing each other (see Fig. 2).

Recovery and PSOC were also described by four participants as promoting the experience of *adding* value to others:

When I started to feel better, I was about to go to The Church City Mission and ask if they needed a hand. When I get better again, I want to do that…most likely, that is what I will do when I have recovered. Make an effort, a volunteering effort in my community. As long as my body can take it.

A similar experience was shared by another participant who in his process of recovery from polysubstance use developed a positive PSOC in the Salvation Army, which again resulted in enhanced self-esteem and a desire to add value to others in his local community. Adding value could be small practical things like janitor services at the institution one resided in, hosting get-togethers for residents, but also more wide-ranging things like “giving back to society”:

…there are a lot of us (with substance use problems) who experience great value in giving back to society…there are people like me who have the need to “OK, I know how you should do that, and I can assist you and help you”. That is important and shouldn’t be ignored.

As such, an enhancing interrelation between recovery processes, PSOC and mattering—both for the dimension of feeling valued by self and others, as well as adding value to self and others—were evident in the older adults’ experiences of recovery from different substance use problems.

## Discussion

Throughout life, we all need to feel valued by, and add value to, self and others. Older adults with substance use problems is a group on a steep rise in several Western societies [21–23]. Thus, there is a pressing need today for substance use services tailored for this group [23–25]. Moreover, in order for substance use services to promote mattering for the many older adults they encounter, there is a need to better understand mattering among older adults receiving substance use services in their recovery processes. The aim of this study was to explore and understand experiences of mattering for older adults in service-assisted recovery processes from substance use problems.

Taken together, the findings show different nuances of experiences of mattering and not mattering (see Table 2). Moreover, the findings reflect several previous findings of later life mattering: that both mattering and not mattering are central parts of older adults’ experiences [11, 13, 19]; that belonging and mattering are distinct but interrelated concepts for older adults [19, 20]; that mattering and a sense of being seen are important in later life recovery processes [63]; and that loss of mattering (by death of e.g. spouse) and capacity (by declining health) are particular features of later life mattering [19].

**Table 2:** Overview of themes and sub-themes

Theme 1: Relational experiences of mattering and not mattering
Family, partner and friend-related experiences of mattering
Family-related experiences of not mattering
Theme 2: Service-related experiences of mattering and not mattering
Being respected, understood and seen
Being ignored and neglected
Theme 3: Recovery, psychological sense of community and mattering as interrelated phenomena
Interrelations in feeling valued by self and others
Interrelations in adding value to others

The findings also provide some new insights. One of the overarching themes in the material was about later life mattering as an interrelated and multidimensional process. Mattering seemed to evolve in a reciprocal interrelation with recovery and relational PSOC: having multiple sources (e.g. family, and services) and including experiences of both mattering and not mattering. Furthermore, the findings provide insight to elements of adding value to others, which prior research on later life mattering rarely address. The participants’ desire to add value to others seemed to be promoted by PSOC and recovery, once again indicating an enhancing interrelation between PSOC, recovery and mattering. These new insights are in line with prior and more general proposals of mattering as a multidimensional concept, co-created in multiple communities [64].

Furthermore, the findings nuance the current understanding of later life capacity of mattering by suggesting that this aspect also includes *social* resources. Although health and illness management are important ingredients to later life capacity in recovery processes, later life capacity is not only about individual ability to matter, but also about the available social resources at hand to enable courage to live and the capacity to matter. Thus, the findings support an understanding of recovery as a relational [31] and social [37] process. Moreover, they indicate that mattering may be a central and fruitful concept for understanding additional aspects of relational and social conceptualizations of recovery [31, 35–37], as well as PSOC for older adults recovering from substance use problems.

Finally, we want to underline the important observation that there was an overall experience of not mattering among the participants (see Fig. 1, stage 2). Several of the participants’ accounts were about experiences of *not* mattering; promoted by relational communities’ lack of support, disrespect and devaluation and loss of relationships due to death, but also being ignored and not receiving fair treatment and help by service professionals. As such, the findings suggest that some older adults with substance use problems may be particularly subjected to the negative effects of not mattering, such as suicidal ideation and attempts [10–13], depression [14, 15], loneliness [17], overall stress [18], marginalization and experiences of being excluded, devalued, and disrespected [2]. Thus, extensive efforts should be initiated to promote the therapeutic and preventive potential of later life mattering for the growing population of older adults with substance use problems.

### Practical implications

Consistent with the findings of this study, we suggest that older adults in service-assisted recovery processes should be supported and helped in staying connected to family and friends able to facilitate their mattering. Network meetings and the involvement of significant others in treatment are concrete examples for how substance use services can include sources of mattering for older adults in recovery. In these meetings and involvements it is important that service providers assist the older adults in clearly communicating what they need from family and friends, and clearly asking family and friends what they can do. The findings, moreover, suggest that mattering is enhanced through help, respect and reciprocal satisfaction of needs. We can say that mattering, PSOC and recovery are co-created. This is where friends and family come into play with the recovering individual and his/her services. A related central task for service professionals is to balance the fine line between experiences of mattering and not mattering in the involvement of relational communities. Promoting the potential enhancing interrelation between recovery, psychological sense of community and mattering is another related key task in this interplay.

Our findings also indicate that several service-related experiences promote experiences of *not* mattering (e.g., experiences of being ignored and neglected), which can be destructive to older adults’ recovery [13, 63]. Thus, when it comes to service-providers, it is essential to make the recovering person feel welcomed, understood and seen, that they their problems and needs matter, are recognized and prioritized in treatment and in follow-up. Furthermore, the findings indicate that some older adult service users may be in particular risk of losing community belonging and mattering in everyday life (e.g. due to loss of partner, loss of friends or decline in health and mobility). Increased investment of professionals’ assistance in service users at the time of such psychosocial transitions can be a crucial step to prevent experiences of not mattering. In example, professionals may provide assistance in grief. Also, to follow-up, one may alter the service users’ individual care plan together with the service user, so that it includes new relevant sources for the experience of feeling valued and adding value to self and others.

Furthermore, the participants’ descriptions suggests that older adults living and recovering in their home need additional service options that can fulfil their basic social needs. We advise that outreach services such as Flexible Assertive Community Treatment (FACT) Teams tailored for older adults should be strengthened and offered more widespread. Such services are crucial when health policy, for example in Norway, is structured around the ideal of keeping older adults in their home for as long as possible. Such services also become increasingly important to assist other services in securing the social needs, health and sense of mattering of the growing population of older adults with substance use problems.

Directorates of health, municipal health services organizations and society at large also have roles to play. These organizations can institute preventive and promoting policies, programs and practices on mattering among recovering older individuals. Training of service providers (e.g. home nurses, low threshold services, geriatric psychologist, and FACT team professionals) in the promotion of later life mattering, and developing programs where recovering service users can feel valued, but also add value to others, are central steps to improve public health. Our findings suggest that receiving support from service professionals having competence for identifying older adults’ needs in their recovery, can be important to promote the experience of service-related mattering. Consistent with the literature about user involvement and shared decision making when it comes to mattering for service providers [40–43], and recovery as a relational and social process [31, 37], it is essential that such policies, programs and practices build partnerships with service users and afford them an opportunity to express their wishes and desires to matter and to be an active part of the decision-making processes affecting their treatment and their lives. The more service users feel understood, empowered and part of their recovery process, the more likely they are to feel like they matter in the eyes of professionals and service providers.

Society also needs to build communities and social networks more systematically around older adults in their recovery. This may entail providing affordable housing and the nourishment of supportive communities, where each person can have a meaningful role. Finally, municipalities may develop Asset Based Community Development (ABCD) programs [65] for promoting mattering between generations of citizens, including older adults in recovery from substance use problems. As our findings also suggest that several older adults in service-assisted recovery processes have a desire to be part of and use their assets to add value to their community and society. Thus, policy and communities can make sure that these potential assets come to life through tailored programs and available sources of mattering.

### Strengths, limitations and future research

There are several strengths and limitations to this exploratory study. First, one of the greatest strengths of the study is the collaborative and multi-perspective approach to the analysis. This approach included nuances of a community psychological, sociological as well as a peer perspective, and is likely to have enhanced reflexivity and interpretive depth through the process of the analysis. On the other hand, the theoretical and deductive approach of the analysis may have restricted the understanding of participants’ concepts of mattering to the current theoretical framework and the researchers’ perspectives (for further detail, see Publisher's Note). To advance a valid understanding of older adults’ experiences, future analyses should consider the possibility of exploring the subject from additional relevant scientific perspectives in deductive analyses, including the perspective of older adult peer researchers (see [66] for suggestions). Future studies should also consider using inductive approaches.

Secondly, another strength of the study is that it includes a rather heterogeneous sample of older adults with different substance use problems. However, consistent with the constructivist perspective, the experiences shared by the participants are the reality of the participants and most probably cannot be transferred to the larger population. Furthermore, we were not able to include more than 7 women with alcohol and medicine problems. Most likely, this restricted our understanding of nuances in older adult women’s experiences of mattering and recovery from these two substance use problems. It also means that the experiences of older adult women recovering from illegal substance use problems were not included in the material. To understand concepts of mattering among older adults in recovery more broadly, future studies should conduct additional qualitative explorative studies including samples representing the demographic profile of the population.

 Given that service systems and social-cultural contexts may differ greatly and affect concepts of mattering across the world, it is important that future studies are planned and undertaken in a systematically context-sensitive way.

## Conclusions

We have investigated experiences of mattering among older adults in service-assisted recovery processes from different types substance use: alcohol, medication and illegal substances. Taken together, experiences of mattering for the participants seem to depend on fair, healthy and positive social relationships and fair and experiences of attentive services, where they can feel valued and have a chance themselves to add value to their own and others’ lives though their recovery processes. Furthermore, the findings suggest that later life mattering must be understood with respect to the older adult life stage, with particular features and psychosocial transitions (e.g. loss of mattering due to death of partner and friends). Importantly, reciprocal and enhancing relations between mattering experiences, recovery and relational PSOC seem to exist: both for the dimension of *feeling valued* by self and others and of *adding value* to self and others. These interrelations also seemed to be significant for the older adults’ access to substance use services and recovery. We have suggested several approaches to promote mattering for older adults in service-assisted recovery processes. At this point, however, more evidence about later life mattering is strongly needed. Additional research, policies, programs and practices are needed to develop and co-create services tailored for promoting mattering and preventing experiences of not mattering for the growing populations of older adults with substance use problems, in their relationships and their communities across the world.

## Data Availability

All documents from the analysis are available upon request from the corresponding author. Due to ethical and privacy concerns for the participants, the full data set is not available.

## Footnotes

1 See “Publisher's Note”.

2 There were similar descriptions by participants having problematic use of illegal substances.

3 P is an abbreviation for participant and I for interviewer.
